# Automated Lobe-Based Airway Labeling

**DOI:** 10.1155/2012/382806

**Published:** 2012-10-09

**Authors:** Suicheng Gu, Zhimin Wang, Jill M. Siegfried, David Wilson, William L. Bigbee, Jiantao Pu

**Affiliations:** ^1^Department of Radiology, University of Pittsburgh, Pittsburgh, PA 15213, USA; ^2^Department of of Pharmacology & Chemical Biology, University of Pittsburgh, Pittsburgh, PA 15213, USA; ^3^Department of Medicine, University of Pittsburgh, Pittsburgh, PA 15213, USA; ^4^Department of Epidemiology, University of Pittsburgh, Pittsburgh, PA 15213, USA; ^5^Department of Bioengineering, University of Pittsburgh, Pittsburgh, PA 15213, USA

## Abstract

Regional quantitative analysis of airway morphological abnormalities is of great interest in lung disease investigation. Considering that pulmonary lobes are relatively independent functional unit, we develop and test a novel and efficient computerized scheme in this study to automatically and robustly classify the airways into different categories in terms of pulmonary lobe. Given an airway tree, which could be obtained using any available airway segmentation scheme, the developed approach consists of four basic steps: (1) airway skeletonization or centerline extraction, (2) individual airway branch identification, (3) initial rule-based airway classification/labeling, and (4) self-correction of labeling errors. In order to assess the performance of this approach, we applied it to a dataset consisting of 300 chest CT examinations in a batch manner and asked an image analyst to subjectively examine the labeled results. Our preliminary experiment showed that the labeling accuracy for the right upper lobe, the right middle lobe, the right lower lobe, the left upper lobe, and the left lower lobe is 100%, 99.3%, 99.3%, 100%, and 100%, respectively. Among these, only two cases are incorrectly labeled due to the failures in airway detection. It takes around 2 minutes to label an airway tree using this algorithm.

## 1. Introduction

 Airway disease is a major cause of morbidity and mortality worldwide [1, 2]. Whereas the airways are the primary conductive structure of the human respiratory system for exchanging air, their morphological characteristics or variations (abnormalities) may have a direct impact on the airflow, thereby alternating the pulmonary function and the prognosis of lung diseases (e.g., chronic obstructive pulmonary disease, (COPD)). Given its high temporal and spatial resolutions, modern computed tomography (CT) makes it possible to noninvasively identify *in vivo* airway structures and quantify their variations. In the past decades, there have been numerous methods [3–6] developed for computerized airway analyses as a way of investigating the underlying mechanism of lung diseases. A relatively comprehensive review can be found in [7]. However, majority of the efforts are dedicated to the global assessment of airway abnormalities at the level of the entire lung. Actually, in anatomy, the human lungs are divided by oblique (or major) and horizontal (or minor) fissures into lobes, with three lobes in the right lung and two lobes in the left lung. Both bronchial and vascular systems in each lobe are largely isolated with minimal connections between lobes, and the lobes can be considered as relatively independent function units. In particular, early diseases of several types may begin in and/or be confined to an individual lobe [8]. Hence, it is desirable to perform quantitative analyses of airway abnormalities based on individual lobes, thereby aiding in the assessment of disease state and progression and/or response to specific treatments. An intuitive approach to this problem is to identify the lobes in advance and then label airway branches according to their locations. However, robust lobe segmentation itself is a relatively challenging issue in practice [9–12]. Therefore, to avoid the complicated lobe segmentation, a straightforward way is to label the airways directly according to their locations in individual lobes. 

 In the past decade, given the fact that manually labeling the airway is very tedious and error prone, a very limited number of computerized approaches [13–17] have been developed to label the airways anatomically. Their primary aim is to assign the airways with the 32 predefined anatomical names, which correspond to different lung segments. Such a labeling may aid the navigation of the airway tree in the virtual bronchoscope application and enable a comparison of the differences between two airways trees that may be acquired on the same or different subjects. In technique, the available labeling approaches were largely implemented using a tree-like structure matching operations. Different variations of graph theory were typically used. For example, Tschirren et al. [13] developed a hierarchical approach based on an association graph to perform the tree matching; Kitaoka et al. [14] implemented the tree matching by searching for the maximum weight clique in a tree association graph (TAG) or subtrees with maximum similarity; Graham et al. [15] proposed a graph-theoretic approach to match airway trees using a cost function that compares branch and branch points measures; van Ginneken et al. [16] proposed a recursive scheme to assign airway labels by measuring a probability based on orientation, average radius, and angles; Feragen et al. [17] presented a supervised hierarchical scheme to label airway trees based on geodesic distance in a geodesic tree space. 

 Obviously, accurately and anatomically labeling the airways is a very challenging task because of anatomical variations across individuals and the large amount of airway branches contained in an airway tree. In this study, instead of labeling the airways at the level of lung segment, we describe an approach that is capable of directly labeling the airways in terms of lobes without the involvement of lobe segmentation. The purpose is to aid regional lobe-based abnormality analysis and other clinical applications (e.g., surgical planning). A robust skeletonization is developed by computing the repulsive force field [18, 19] of an airway tree; thereafter, basic lung anatomy knowledge in regard to airways is used for labeling or classification. Due to the variability in biological structures across individuals and the presence of various diseases, we in particular developed a self-correction mechanism. The performance of the developed scheme was assessed by testing it against an independent dataset consisting of 300 chest CT examinations. A detailed description of the method and the experimental results follows. 

## 2. Methods and Materials

### 2.1. Scheme Overview

**Figure 1:**
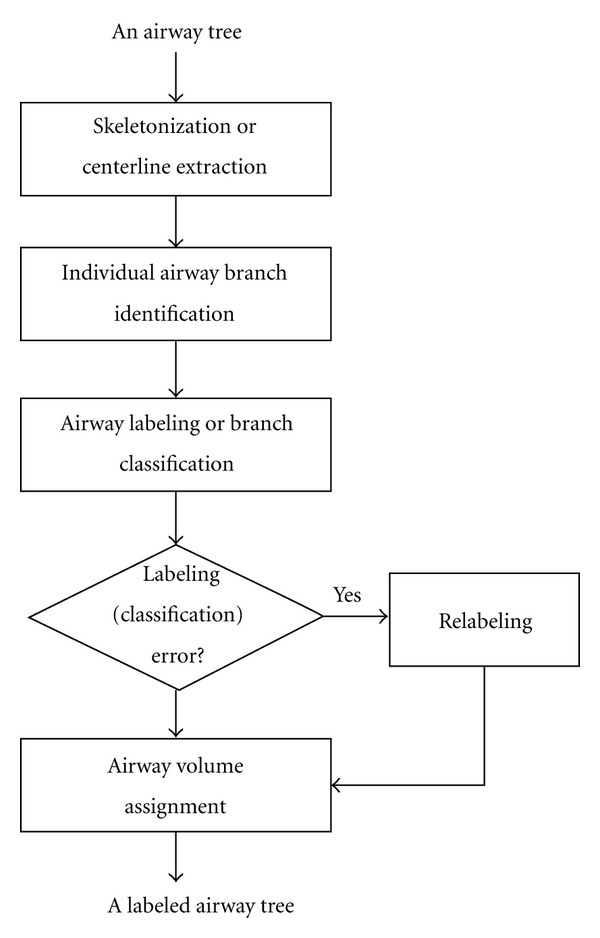
A flow chart illustrating the procedures of the developed airway labeling scheme.

 In implementation, given an airway tree, the proposed airway labeling approach has three basic steps (Figure 1): (1) airway skeletonization or centerline extraction, (2) individual airway branch identification, (3) initial airway labeling or classification in terms of pulmonary lobe, and (4) self-correction of labeling errors. This scheme is independent of airway segmentation schemes. The airway tree identified by any airway segmentation approach can be used as the input of the scheme. In this study, we simply used the airway segmentation scheme described in [20] to obtain the airway trees. For brevity, we use RUL, RML, RLL, LUL, LLL to denote the right upper lobe, right middle lobe, right lower lobe, left upper lobe, and left lower lobe, respectively. Detailed descriptions of these steps follow. 

### 2.2. Airway Skeletonization or Centerline Extraction

Attributed to its concise representation of a shape, skeleton (frequently referred as medical axis) is widely used in computerized shape analysis. The skeletonization is typically associated with intensive computational cost and is sensitive to the existence of noises. In the past, various approaches [21–23] have been developed to obtain the skeleton of an airway tree by taking advantage of its tubular structures. In this study, we optimized a generic algorithm based on repulsive force field developed by Cornea et al. [18] to automatically obtain the skeleton of an airway tree. 

 Given an airway tree that is represented in the form of image voxels, its three-dimensional (3D) vector field is computed using the repulsive force function:

(1) $$\begin{array}{c} \overset{\to }{F_{PC}}=\frac{\overset{\to }{CP}}{R^{m}}, \end{array}$$

where $\overset{\to }{F_{PC}}$ is the repulsive force at point *P* with respect to the point charge *C*, $\overset{\to }{CP}=\left(P-C\right)/R$ is the normalized vector from *C* to *P*, indicating the direction of the force, *R* = ||*P*−*C*||_*F*_ is the Euclidean distance between *P* and the charge *C*, and the power *m* is the order of the force function (*m* = 2 for the Newtonian force and *m* = 4 in this study). As explained in [18], a larger value of *m* typically has a higher influence on a given interior point than on the distance boundary points, ultimately leading to a “sharp” vector field that follows the local boundary topology more closely. On the contrary, a lower value of *m* will result in a smoother vector field. Given the tubular-like structure of the airways in shape, a relatively low value of *m* is preferred because we are only interested in the skeleton of the airways that reflect the global shape of the airways, and the local details with high frequency (e.g., the surface perturbation of the airways) should be ignored. The total force at point *P* can be computed by summing all the forces at *P*:

(2) $$\begin{array}{c} \overset{\to }{F_{P}}=\sum_{i}\overset{\to }{F_{PC_{i}}}, \end{array}$$

where $\overset{\to }{F_{P}}$ is the final repulsive force at point *P* due to all point charges *C*_*i*_. 

**Figure 2:**
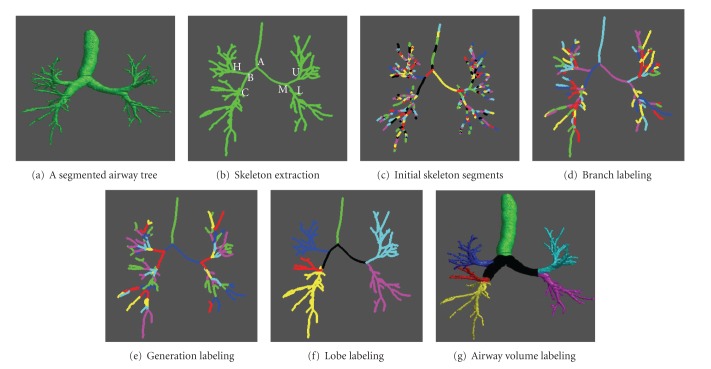
An example used for illustrating the steps of the labeling algorithm: (a) a segmented airway tree that can be obtained using any airway segmentation algorithm, (b) the extracted skeleton/centerline of (a) in the form of point/voxel, (c) skeleton segments obtained by the algorithm of [18], (d) individual branch labeling (in different colors), (e) different generation labeling (in different colors), (f) lobe labeling (trachea, RUL, RML, RLL, LUL, LLL are colored with green, blue, red, yellow, cyan, and magenta, resp.), (g) airway tree volume labeling.

 According to (2), if all the boundary voxels are used to compute the force at each point *P*, the computational complexity will be *O* (*N*_*P*_ × *N*_*C*_), where *N*_*P*_ is the number of object (airway) voxels and *N*_*C*_ is the number of boundary voxels (point charges). Hence, given the large number of the object (airway) voxels, it is very time consuming to compute the repulsive force field of an airway tree. In order to significantly improve the computational efficiency, we propose not to consider all the boundary voxels when computing the repulsive force at a given point *P*, because the airways appear as a tubular shape, and the boundary voxels far away from *P* may contribute little to the repulsive force. Therefore, given an object voxel *P*, only the boundary voxels close to *P* (e.g., less than 25 mm to *P*) are considered to compute the repulsive force at *P*. This strategy significantly reduces the computation cost from an average of 30 minutes to less than 2 minutes with little impact on the accuracy of the skeletonization. An example of the skeletonization is shown in Figure 2(b). 

### 2.3. Individual Airway Branch Identification

**Figure 3:**
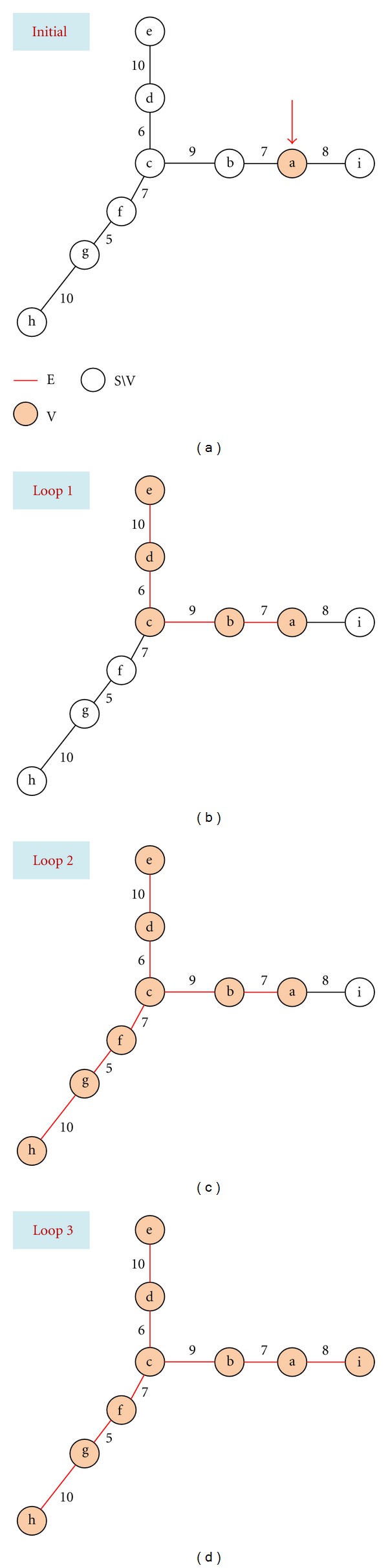
Illustration of the graph construction procedure. Each loop starts with Step 1 and ends at Step 4. In the graph, the nodes (i.e., “a”–“i”) denote the skeleton points, the edges between nodes represent the distance between the skeleton points. In this example, let “a” be the randomly selected point, then other skeleton points are added to the voxel set *V* in the following sequence: “bcde *|* fgh *|* i *|*”, where “*|*” means Step 4 is called.

 The repulsive force field described in [18] can be exploited further to detect the “nodes” by decomposing the identified skeleton into different segments automatically in terms of the divergence of each point on the skeleton. However, the identified segments are sensitive to the curvature of the skeleton, ultimately leading to a number of segments that are not meaningful airway branches (Figure 3(c)). Here we propose to identify individual airway branches by organizing the skeleton points as an undirected acyclic graph (UAG). 

 Let *S* = {*S*_*i*_}_*i*=1_^*N*_*S*_^ denote the *N*_*S*_ extracted skeleton points and *G* = {*V*, *E*} denote a targeting undirected acyclic graph (UAG), where *V* is the vertex set and *E* is the edge set. The graph *G* is initialized with *V* = {*S*_*i*_}, *E* = *ϕ*, where vertex *S*_*i*_ is a point randomly selected from the skeleton points set, and then the following steps are performed.

**Step**

Find a nearest pair of points *S*_*i*_ ∈ *V* and *S*_*j*_ ∈ *S*∖*V*, where *S*_*i*_ has less than three connected neighbors on *G*, let *V* = *V* ∪ *S*_*j*_ and *E* = *E* ∪ (*i*, *j*).

**Step**

Let *S*_*j*_ denote the last inserted point and find its nearest neighbor *S*_*k*_ ∈ *S*∖*V*. If the distance between *S*_*j*_ and *S*_*k*_ is small enough (e.g., smaller than 2 mm), then let *V* = *V* ∪ *S*_*k*_ and *E* = *E* ∪ (*j*, *k*).

**Step**

If a new voxel *S*_*k*_ is added to *V*, go to Step 2; otherwise, go to Step 4.

**Step**

If *S*∖*V* ≠ *ϕ*, go to Step 1; otherwise, the graph construction is finished.

The graph construction algorithm is explained using the example in Figure 3. Once the UAG is constructed, each vertex is connected to one, two, or three other vertexes. A terminate point (e.g., “e”, “h”, or “i” in Figure 3) is connected to only one neighbor point. A bifurcation point (e.g., “c”) is defined as one connected with three neighbor points, thus the branches can be obtained by cutting at the bifurcation points. In Figure 3, the three branches are “cde,” “cfgh,” and "cbai," respectively. An example of skeleton branch decomposition is shown in Figure 2(d).

### 2.4. Initial Airway Labeling or Branch Classification

 To label or classify the identified airway branches, we firstly locate the trachea by identifying the branch with the largest lumen volume. To compute the airway lumen volume of each branch, we assign each airway voxel to its nearest skeleton point and the branch of the skeleton point. Thereafter, the generations of the airway tree are determined by treating the airways as a bifurcation tree (Figure 2(e)).

**Figure 4:**
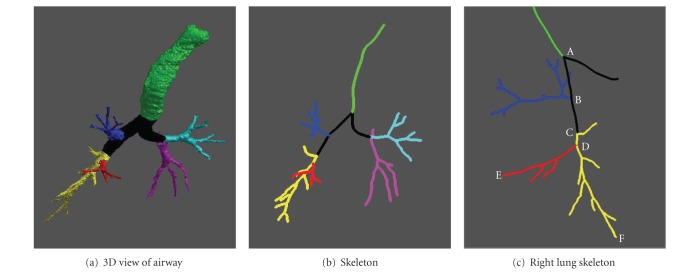
An example of classification of RML and RLL.

Here, basic lung and airway anatomy knowledge is used to classify the individual branches into different categories in terms of pulmonary lobes. First, the left lung and the right lung labels are determined based on *x* values of the two end points (B and M in Figure 2(b)) of the first generation branches. Second, for the left lung, the LUL and the LLL are determined based on the *z* values of the two end points (U and L in Figure 2(b)) of the second generation branches. For the right lung, the RUL is selected with the same criteria by comparing the end points (i.e., H and C in Figure 2(b)). Due to the biological variability across individuals in lung structure, the RML and RLL of the airways are not necessarily divided at the second node of the branches (point C in Figure 4) of the right lung. It can be seen on the sagittal view of the example in Figure 4(c) that the RML is usually at the upper left of the RLL. Using this rule, we select a branch end point E with the maximal value of (*z-y*) and a branch end point F with the minimal value of (*z-y*). Then, the bifurcation node D of point E and F is determined. Finally, the branches between node D and E and their subbranches are labeled as RML, and the other branches of node C are labeled as RLL. An example after the application of the labeling algorithm is shown in Figure 2(f). 

### 2.5. Self-Correction of Labeling Errors

**Figure 5:**
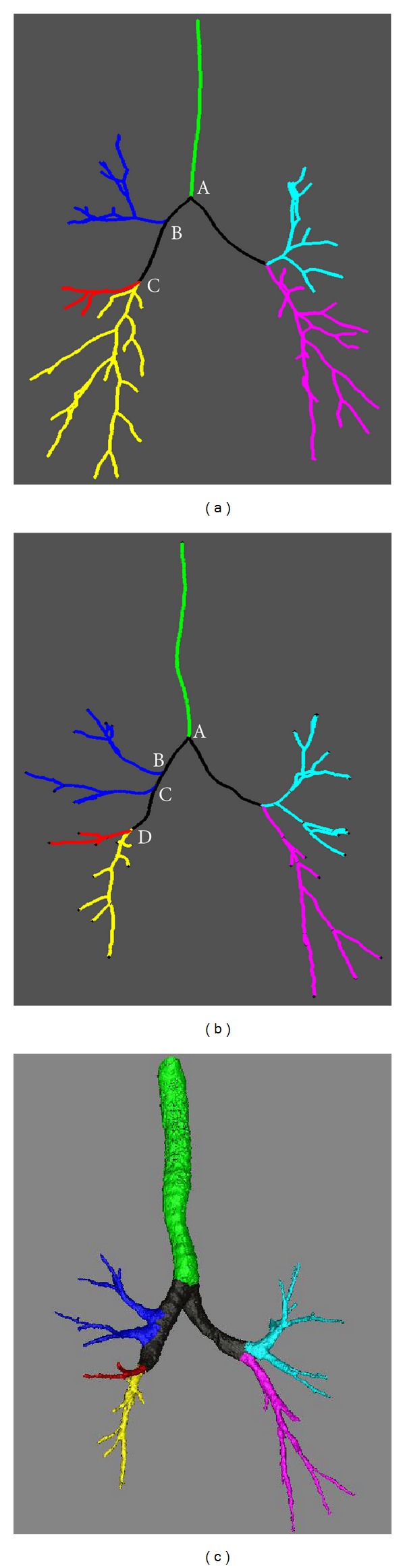
A normal example and an abnormal example. (a) An example with normal topology, (b) an example with abnormal topology, (c) 3D view of the abnormal airway.

 In practice, although the graphs of most airway tree have the same topology in structure, as the one shown in Figure 5(a), there are some exceptions, such as the example in Figures. 5(b) and 5(c). In order to automatically handle such exceptions (errors), we develop a self-correction mechanism. In the example shown in Figures 5(a) and 5(b), A, B, C, D are nodes of the first, second, third, and forth generations. Let AB denote the distance between node A and node B and BC denote the distance between node B and node C. The ratio BC/AB in the exceptional cases (e.g., the example in Figure 5(b)) is typically much smaller than the ratio BC/AB in the normal cases (e.g., the example in Figure 5(a)). This rule typically holds because the two branches in blue after B and C in Figure 5(b) belong to the same lobe (RUL). Hence, we can use this rule to automatically detect the potential labeling error. In other words, if BC/AB is smaller than a given value (e.g., 0.5), the labeling error occurs. As a correction, we label all the branches after nodes B and C as the RUL.

### 2.6. Airway Volume Assignment

 After the airway branches are labeled, the last task is to mark the airway volumetric voxels to each individual branches or lobes. In this study, we assign each airway voxel *P* to its nearest skeleton point S* = arg min⁡_*S*_*i*__⁡||*P*−*S*_*i*_||_*F*_ and the branch of the skeleton point. An example after the application of this airway volume assignment procedure is shown in Figure 2(g). 

### 2.7. Performance Assessment and Testing Dataset

 As demonstrated by the example in Figure 2(g), it is relatively easy to visually examine whether the airways are labeled correctly or not. Hence, in order to assess the performance of the airway labeling algorithm developed in this study, we ask an image analyst to visually study the labeled results and locate the fail ones. Then, the number of the failed cases in terms of lobes is summarized as a way of measuring the accuracy of the airway labeling. 

 The proposed algorithm was evaluated on 300 lung CT examinations specifically from a chronic obstructive pulmonary disease (COPD) dataset available at the University of Pittsburgh Medical Center (UPMC). These CT examinations were performed under an Institutional Review Board (IRB) approved protocol using a LightSpeed VCT 64-detector scanner (GE Healthcare, Waukesha, WI, USA) with subjects holding their breath at end inspiration. CT data were acquired using a helical technique at a pitch of 0.969, 120 kVp, 0.4 s gantry rotation, and 250 mA (100 mAs). The detector configuration was 32 × 0.625 mm. CT images were reconstructed to encompass the entire lung field using the GE “bone” kernel at 0.625 mm section thickness and 0.625 mm interval (without slice overlapping). The CT images were represented using a 512 × 512 pixel matrix with a pixel dimension ranging from 0.549 to 0.738 mm.

## 3. Experimental Results and Discussion

**Figure 6:**
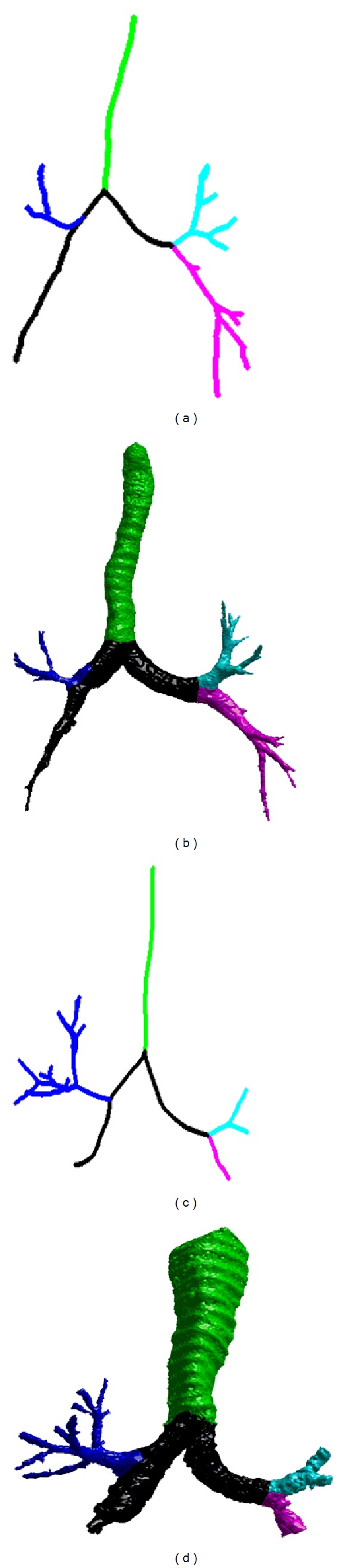
Two failed cases in RML and RLL labeling: (a)-(b) show the skeleton and the labeling result of the first failed example and (c)-(d) show the skeleton and the labeling result of the second failed example.

**Figure 7:**
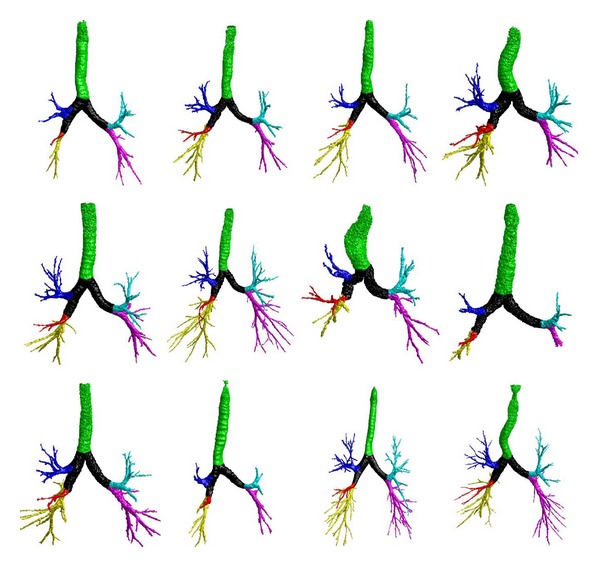
Examples of the airway labeling results.

 We summarized the experimental results in Table 1. Among the 300 chest CT examinations, the labeling operation failed for only two cases (Figure 6) because of the unavailability of the airways in the RML and the RLL. This is actually not caused by the labeling algorithm but by the airway segmentation algorithm. To demonstrate the performance of this scheme, a set of labeled examples are listed in Figure 7. Given an airway tree, the total average computational cost for each case is around two minutes when performing the developed scheme on a typical PC. 

**Figure 8:**
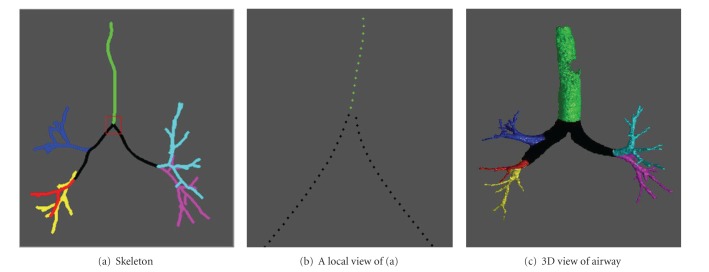
An example where a missing part of the trachea is missed by the airway segmentation scheme.

In this study, we described a simple and efficient algorithm to automatically label the airway trees in terms of pulmonary lobes. The innovations lie in the following aspects. First, the skeletonization is robust, efficient, and accurate regardless of the shape of an airway tree. The chest CT examinations in our testing dataset are randomly selected from a COPD study and cover a wide range of severities in emphysema that has a direct impact on the morphology of airways. Experiments showed that this skeletonization scheme can successfully obtain the centerlines of all these cases in less than two minutes. Second, the airway labeling procedure is performed directly on the skeletons without the involvement of any matching operation like some previous approaches [13–17]. To assign predefined labels to the airway tree, traditional approaches often take a tree-matching procedure, where a prelabeled airway model is used as reference for labeling. However, tree structure matching itself is a challenging problem, especially when two trees have different branch numbers. In particular, we develop a self-correct mechanism to handle the cases where the topology of an airway tree changes. As shown in Figure 6, although there are two cases identified by the image analyst as failed cases, the failure is not caused by the labeling algorithm but by the airway identification procedure. Third, we applied a relatively large dataset to assess the accuracy of the developed scheme. The results show that the proposed airway skeletonization and labeling scheme are accurate and robust. As demonstrated by an example in Figure 8, the airway skeletonization and labeling are correct in spite of the missing of a large part of the airway trachea. 

**Figure 9:**
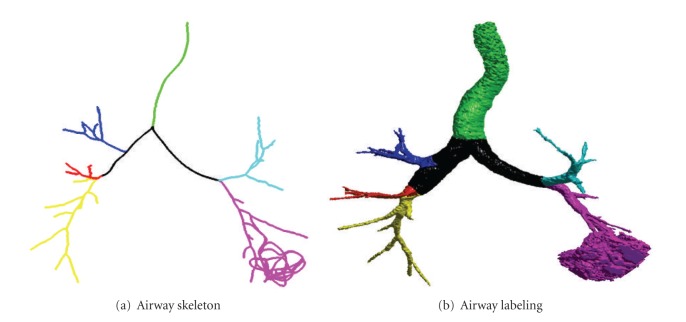
An example with airway leakage.

We are aware that there are some limitations with this study. First, unlike the previous approaches [13–17], which assigned the airways anatomically into 32 names in terms of lung segments, the airway branches are only labeled here in terms of lobes and generations. For more detailed investigation of airway diseases, it may be desirable to have airway labeled at the level of lung segments. In technique and implement, we admit that the lobe-based labeling is relatively easier as compared with the previous approaches. Given the different levels of detail in labeling, it is not proper to compare the performance of our scheme with other available methods. Second, although 300 cases are used to test the performance of this scheme, we cannot guarantee that this dataset covers all types of airway tree topology because of the large variations of human subjects in anatomy. In this study, we only identified one exception as shown in Figure 5(b). In the future, we will collect more dataset from various sources to test the performance of this labeling algorithm. In particular, we asked an image analyst to visually assess the performance of the labeling scheme, because it is relatively easy to visually judge the accuracy and correctness of the lobe-based labeling results given the distinctive boundary of individual lobes (as demonstrated by the examples in Figure 7). In contrast, it is very difficult even impossible to visually judge the accuracy of the tradition methods because of the huge number of airway branches in an airway tree and their self-occlusion. Third, although this algorithm is independent of the airway segmentation scheme in theory and implementation, we do not verify this in practice. As compared with the traditional airway labeling approaches, assigning airways into different lobes is much easier and more robust, because only the first few generations of the airways are involved. As the airway segmentation issues typically occur in the small airway regions [24], we believe that the performance of the airway segmentation scheme (e.g., the leakage issue in the traditional region-growing-based airway segmentation in Figure 9) may have a very limited impact on the lobe-based airway labeling. 

## 4. Conclusion

 We described a simple and efficient algorithm for automated airway labeling in term of pulmonary lobe. The performance of this algorithm is assessed using a large dataset consisting of 300 chest CT examination selected from a COPD study at our institute. Experiments showed that this algorithm is efficient, accurate, and robust. In the future, we will extend this work to label an airway tree at the level of lung segments and collect a larger dataset for assessment purpose. 

## Figures and Tables

**Table 1:** Airway labeling accuracy in terms of lobes.

	Trachea	RUL	RML	RLL	LUL	LLL
Correct #	300	300	298	298	300	300
Percentage	100%	100%	99.3%	99.3%	100%	100%
